# Fentanyl Versus Dexmedetomidine for the Prevention of Emergence Agitation in Children After Sevoflurane Anaesthesia: A Comparative Clinical Study

**DOI:** 10.7759/cureus.28587

**Published:** 2022-08-30

**Authors:** Syeda Parbin Sultana, Diganta Saikia, Sandeep Dey

**Affiliations:** 1 Anaesthesiology, Tezpur Medical College, Tezpur, IND; 2 Anaesthesiology, Assam Medical College and Hospital, Dibrugarh, IND; 3 Neuroanaesthesia, Paras Hospital, Gurugram, IND

**Keywords:** paediatric anaesthesia, emergence agitation, sevoflurane induction, dexmedetomidine, fentanyl

## Abstract

Introduction

Sevoflurane is widely used in pediatric anesthesia due to its rapid onset and offset of action, smooth induction, and less hepatotoxicity. However, it is associated with emergence agitation, which can be frightening and harm the patient or the caregiver. While a definite preventive measure of emergence agitation is in search, the use of some drugs is associated with a lesser incidence of emergence related to sevoflurane.

Aims and objective

This study aimed to compare the efficacy of fentanyl with dexmedetomidine in preventing emergence agitation in children undergoing surgery with sevoflurane anesthesia. Also, we assessed the perioperative hemodynamic and postoperative recovery characteristics and side effects, if any, between children receiving the two groups of drugs.

Material and method

We conducted a prospective, double-blinded, randomized controlled trial after getting approval from the institutional ethical committee. A total of 120 patients were recruited into the study and divided into two groups, F and D, of 60 patients each. Patients in group F received an infusion of injection fentanyl at 1 mcg/kg and patients in group D received infusions of injection dexmedetomidine at 0.15 mcg/kg, respectively, after induction of general anaesthesia. Additionally, all patients received a caudal epidural block with 0.125% isobaric levobupivacaine. After the conclusion of surgery, we transferred the patients to the post-anaesthesia care unit for further observation and assessment.

Result

The Pediatric Anesthesia Emergence Delirium (PAED) score for emergence agitation was significantly greater in the fentanyl group compared to the dexmedetomidine group at 0 minutes (7.08 ± 1.03 vs. 6.43 ± 1.33, p = 0.003) and 15 minutes (5.51 ± 1.7 vs. 4.01 ± 1.08, p = 0.0001) postoperative period. The mean time to eye-opening was significantly earlier amongst children receiving fentanyl than those receiving dexmedetomidine (9.3 ± 1.1 min vs. 10.12 ±1.05 min, p = 0.0001). The modified Aldrete Score for adequacy of recovery was statistically insignificant initially, but as the duration progressed to 15 minutes, the children in the fentanyl group had significantly higher scores than those in the dexmedetomidine group (8.05 ± 0.67 vs. 7.76 ± 0.62, p = 0.01).

Conclusion

Prophylactic administration of dexmedetomidine (0.15 mcg/kg) or fentanyl (1 mcg/kg) administered is effective in preventing emergence agitation. Although we found emergence agitation was higher amongst children receiving fentanyl than those receiving dexmedetomidine during the early recovery period, this difference became insignificant as the postoperative period increased to 30 minutes.

## Introduction

Inhaled induction of anaesthesia is a commonly used method by pediatric anaesthesiologists [1]. It is an essential technique in situations such as lack of venous access and anticipated airway difficulty. Halothane, sevoflurane and desflurane are the anaesthetics of choice for the inhalational induction of anaesthesia in children [2]. Sevoflurane is widely used in pediatric anaesthesia owing to its rapid action, relative lack of airway irritation, low hepatotoxicity, hemodynamic stability, and rapid emergence from anaesthesia [3,4]. However, sevoflurane anaesthesia in children is associated with a higher incidence of emergence agitation than halothane or desflurane [2]. Emergence agitation usually occurs within the first 30 minutes of recovery from anaesthesia. Although mostly self-limiting (5-15 min), it can be frightening for the parents or hospital staff. There may be physical harm to the child and can dislodge intravenous catheters, dressings, access, and drains [5]. While we are yet to identify a definite preventive measure, the literature suggests a lower incidence of emergence agitation with the use of pharmacological agents such as midazolam, clonidine (orally or epidurally), ketorolac, fentanyl, or dexmedetomidine (intravenously) [5-7].

To offer pediatric patients a more comfortable anaesthesia experience without emergence agitation, we conducted a prospective trial wherein our primary objective was to compare the efficacy of fentanyl with dexmedetomidine in preventing emergence agitation in children undergoing surgery with sevoflurane anaesthesia. Our secondary objective was to assess the perioperative hemodynamic and post-operative recovery characteristics and side effects, if any, between the two groups.

## Materials and methods

After obtaining approval from the Institutional Ethical Committee (registration number: ERC/636/Inst/AS/2014; approval number: AMC/EC/PG 12494; dated March 3, 2017), we conducted a prospective, double-blinded, and randomized controlled trial from July 2017 to June 2018 in Assam Medical College and Hospital, in the state of Assam, India. We obtained written and informed consent from the parents of each child after explaining the study procedure to them in their language. All 120 patients of either sex between the ages of two to eight years, belonging to American Society of Anaesthesiologists (ASA) grades I and II, and scheduled for elective sub-umbilical surgery were included, and patients for whom we couldn't obtain consent, patients with ASA grade III or higher, those with cognitive impairment, coagulation disorders, allergy to any of the study drugs, those requiring sedative premedication, and who were undergoing emergency surgery were excluded.

**Figure 1:**
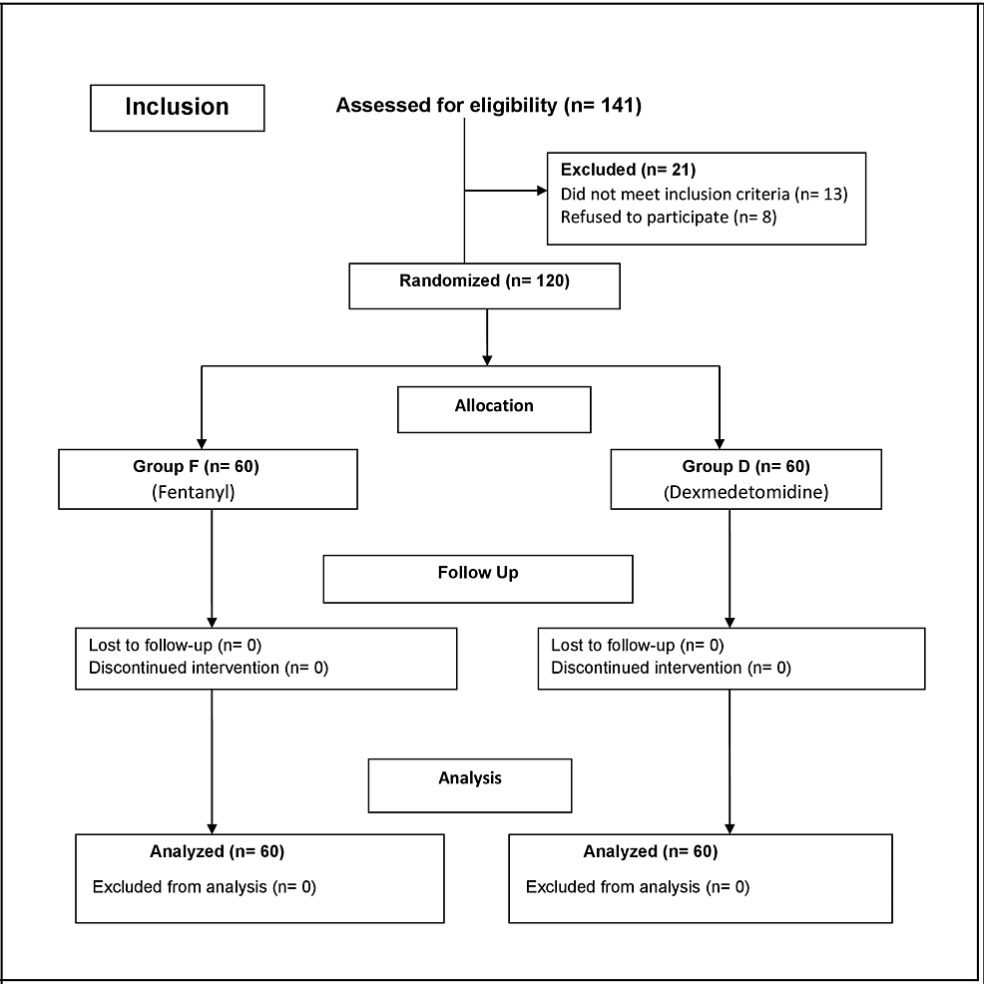
CONSORT flow diagram CONSORT: Consolidated Standards of Reporting Trials

We randomly divided the patients into two groups using a concealed envelope method. Each group consisted of 60 children; one group was named Group F (patients receiving fentanyl) and the other, Group D (patients receiving dexmedetomidine) (Figure 1). A senior nurse confirmed the patient's identity and data in the pre-operative holding area. After bringing the patient into the operation theatre (OT), all ASA standard noninvasive monitors, including pulse oximetry, electrocardiogram, noninvasive blood pressure, and skin temperature probes, were attached. General anaesthesia was induced with 50% nitrous oxide (N2O) and 50% oxygen (O2) with 8% sevoflurane in a single-step manner. After the patient lost consciousness and attained an adequate depth of anaesthesia (after two to three minutes of unconsciousness), we cannulated a peripheral vein with an intravenous (IV) catheter of appropriate size for drug and fluid administration. Afterwards, anaesthesia was maintained by varying the sevoflurane concentration between 1-3% in 50% N2O and 50% O2 until the end of surgery. All patients received an injection of glycopyrrolate at a dose of 4mcg/kg to all children after placement of the IV catheter.

Then we infused the study drugs (injection fentanyl at 1 mcg/kg in group F and injection dexmedetomidine at 0.15 mcg/kg in group D) over 10 minutes. To ensure blinding, an independent senior nurse not involved in the study prepared the study drugs in a total volume of 10 ml of normal saline.

After achieving the adequate depth of anaesthesia, a supraglottic airway device, i.e., i-Gel (Intersurgical, Wokingham, United Kingdom), of appropriate size for the child's weight, was placed for intra-operative maintenance of a patent airway. All the children were maintained on assisted ventilation via a Jackson Rees circuit until the end of the procedure. A caudal epidural block was performed with 0.125% isobaric levobupivacaine (0.5ml/kg volume) to combat perioperative pain in both groups' children. We used lactated ringer's solution in 5% dextrose according to the patient's body weight, fluid deficit, and surgical loss (as per the Holliday Segar rule). Sevoflurane and N2O were discontinued before the last stitch was over. Before the patient was awake, the i-Gel was removed and the time to eye-opening was calculated (defined as the time from the end of sevoflurane anaesthesia to eye-opening on command).

After transferring the patients to the post-anaesthesia care unit (PACU), an assistant blinded to the patient assignment observed the subjects in PACU and documented the agitation using the Paediatric Anesthesia Emergence Delirium scale (PEAD scale) at 0, 15, and 30 minutes [8]. The observer evaluated the pain by Children's Hospital of Eastern Ontario Pain Scale (CHEOPS) score at 0, 15, and 30 min, and adequacy of recovery by Modified Aldrete score at 0, 15, and 30 min [9,10]. In the PACU, fentanyl 0.5 mcg/kg was administered for a CHEOP score of 10 if the patient requested pain medication or cried during two consecutive scoring periods. Patients with PAED scores≥11 were managed with IV propofol 1 mg/kg. We also recorded the duration of surgery, time to eye-opening and the time from eye-opening to discharge from PACU, and instances of adverse events, if any, during the perioperative period.

Sample size calculation

Assuming the level of significance (α) as 0.05 and the power of the study as 0.90 (β = 0.10), and in an attempt to detect an effect size of at least 0.4, concerning the primary study outcome variable (the PAED score), we found that 108 patients would be needed to answer our research question. Considering a 10% chance of loss to follow-up, we have included 120 patients, with 60 patients in each group, by rounding off our study population.

Randomization

An independent senior resident divided the study participants into two equal groups of 60 patients, each using the computer-generated random number table. We allocated the patients into Group F (who received fentanyl infusion) and Group D (who received dexmedetomidine infusion). A senior nurse not involved in the study used the SNOISE (sequentially numbered, opaque, sealed, and stapled envelopes) method to conceal the allocation process from the investigator while enrolling the participants into the study groups. Another independent senior nurse opened the envelope, prepared the study drug as mentioned in the envelope in a standard volume of 10 ml, and handed it over to the anaesthesiologist performing the study. The anesthesiologist recorded the patient and result variables on a piece of paper and handed it over to the senior nurse, who secured and stored it along with the envelope in a secure place for further analysis by the principal investigator.

Statistical analysis

SPSS statistical software (version 20.0; IBM Corporation, Armonk, NY) was used for data analysis and computation. We presented the numerical data (e.g., age, blood pressure, CHEOPS score, PAED score, modified Aldrete score) as mean and standard deviation and categorical data (e.g., sex) as frequency (%). We used the Student's t-test for numerical variables and the chi-square test for categorical variables to assess the statistical significance. A p-value of <0.05 was considered statically significant.

## Results

Overall, 141 children were assessed of which 120 were included in the study (Figure 1). Table 1 demonstrates the demographic characteristics. We found that both the groups were comparable and there was no statistically significant difference in the demographic parameters amongst them. Table 2 demonstrates the different surgical procedures the participants in the two groups underwent which consisted of mainly circumcision, orchidopexy and inguinal hernia repair. Table 3 demonstrates the duration of anaesthesia (in minutes) amongst the different participants in both groups. We found no statistical difference between the two groups.

**Table 1:** Demographic parameters F, fentanyl; D, dexmedetomidine; SD, standard deviation. * Student's t-test, ** chi-square test.

Variable	Group		P value
	F (n = 60)	D (n = 60)	
Age (years), mean ± SD	5.02 ± 1.77	5.23 ± 1.75	0.51*
Weight (kg), mean ± SD	15.68 ± 3.48	16.05 ± 3.50	0.56*
Sex, n (%)			
Male	43 (71.7)	39 (65)	0.43 **
Female	17 (28.3)	21 (38)	

**Table 2:** Type of surgeries F, fentanyl; D, dexmedetomidine.

Surgical procedure	Group F		Group D	
	n	%	n	%
Circumcision	22	36.67	25	41.67
Inguinal hernia repair	32	53.33	30	50
Orchidopexy	6	10	5	8.33
Total	60	100	60	100

**Table 3:** Duration of anaesthesia F, fentanyl; D, dexmedetomidine. *Student's t-test.

Duration of anaesthesia (minutes)	Group F		Group D		P value
	N	%	n	%	1*
0 – 40	2	3.33	1	1.67	
40 – 50	49	81.67	47	78.33	
50 – 60	9	15.00	12	20.00	
Total	60	100	60	100	

Table 4 gives a comparative evaluation of the hemodynamic variable (heart rate) between the two groups at different time intervals. The pre-operative (baseline) heart rates between the groups were comparable. The heart rate significantly decreased in Group D compared to Group F at 0 minutes, 10 minutes and 20 minutes while towards the later part of the intraoperative period i.e. at 30 minutes and 40 minutes the decrease in heart rate was insignificant and hence comparable.

**Table 4:** Hemodynamic data (heart rate) F, fentanyl; D, dexmedetomidine; SD, standard deviation. *Student's t-test.

Time		Heart rate (beats/minute)		p value*
		Group F (mean ± SD)	Group D (mean ± SD)	
Pre operative		107.57 ± 13.25	106.42 ± 12.26	0.62
Intra operative	0 min	97.65 ± 11.29	87.9 ± 10.89	0.0001
	10 min	98.33 ± 11.46	90.11 ± 11.09	0.0001
	20 min	99.15 ± 11.35	92.08 ± 10.81	0.0007
	30 min	100.53 ± 11.44	97.03 ± 11.19	0.09
	40 min	100.97 ± 11.29	98.9 ± 10.9	0.3
Post operative	0 min	101.2 ± 11.27	99.9 ± 10.8	0.52
	15 min	103.5 ± 11.89	102.65 ± 11.36	0.68
	30 min	105.7 ± 12.51	104.4 ± 12.11	0.56

Table 5 demonstrates the change in blood pressure in the two groups at different time intervals. The pre-operative mean arterial pressure (MAP) between the groups was comparable. The MAP significantly decreased in Group D compared to Group F at 0 minutes, 10 minutes and 20 minutes, while later at 30 minutes and 40 minutes the decrease in MAP was statistically insignificant.

**Table 5:** Hemodynamic data (blood pressure) MAP, mean arterial pressure; F, fentanyl; D, dexmedetomidine; SD, standard deviation. *Student's t-test.

Time		MAP (mmHg)		p value*
		Group F (mean ± SD)	Group D (mean ± SD)	
Pre-operative		78.88 ± 9.97	77.03 ± 7.9	0.26
Intra-operative	0 min	72.66 ± 9.35	68.9 ± 6.68	0.01
	10 min	74.03 ± 9.21	70.38 ± 6.8	0.01
	20 min	74.96 ± 9.4	71.7 ± 7.07	0.03
	30 min	77.43 ± 9.98	74.7 ± 7.88	0.09
	40 min	77.98 ± 9.9	75.97 ± 7.9	0.22
Post-operative	0 min	78.42 ± 9.77	76.17 ± 7.88	0.16
	15 min	78.56 ± 9.78	76.48 ± 7.77	0.19
	30 min	78.92 ± 9.89	76.77 ± 7.77	0.18

Table 6 gives the mean time to eye-opening after stopping the inhalational agents, and it was significantly earlier in Group F compared to Group D. Table 7 gives the mean time of shifting to the ward after removal of the I-gel. It was also found to be significantly earlier in Group F compared to Group D.

**Table 6:** Time to eye-opening F, fentanyl; D, dexmedetomidine; SD, standard deviation. *Student's t-test.

Group	Time (in minutes) (mean ± SD)	p value
F	9.3 ± 1.1	0.0001*
D	10.12 ± 1.05	

**Table 7:** Time taken to shift to ward F, fentanyl; D, dexmedetomidine; SD, standard deviation. *Student t-test.

Group	Time (in minutes) (mean ± SD)	p value
F	17.99 ± 1.95	0.0001*
D	19.38 ± 1.63	

Table 8 gives the CHEOPS score of the two groups. To prevent pain as a confounding factor, we used the caudal block. The pain was statistically significant between the two groups at 0 minutes, with Group D having higher scores than Group F. But later, from 15 minutes to 30 minutes postoperatively, the pain scores between the two groups became insignificant. Table 9 gives the modified Aldrete score for adequacy of recovery. The scores were insignificant at 0 minutes. By 15 minutes group F had a significantly higher score, but by 30 minutes the scores were insignificant. Table 10 gives the PAED score for emergence agitation. It was significantly greater in Group F compared to Group D at 0 minutes and 15 minutes. However, by 30 minutes, the scores were comparable and statistically insignificant.

**Table 8:** CHEOPS score F, fentanyl; D, dexmedetomidine; SD, standard deviation; CHEOPS, Children's Hospital of Eastern Ontario Pain Scale *Student's t-test.

Post-operative period	Modified Aldrete Score		p value*
	Group F (mean ± SD)	Group F (mean ± SD)	
0 min	7.13 ± 0.7	6.93 ± 0.7	0.12
15 min	8.05 ± 0.67	7.76 ± 0.62	0.01
30 min	10 ± 0	10 ± 0	-

**Table 9:** Modified Aldrete score F, fentanyl; D, dexmedetomidine; SD, standard deviation. *Student's t-test.

Post operative period	Modified Aldrete Score		p value*
	Group F (mean ± SD)	Group F (mean ± SD)	
0 min	7.13 ± 0.7	6.93 ± 0.7	0.12
15 min	8.05 ± 0.67	7.76 ± 0.62	0.01
30 min	10 ± 0	10 ± 0	-

**Table 10:** PAED score F, fentanyl; D, dexmedetomidine; SD, standard deviation; PAED score, Pediatric Anesthesia Emergence Delirium score *Student's t-test.

Post operative period	PAED Score		p value*
	Group F (mean ± SD)	Group F (mean ± SD)	
0 min	7.08 ± 1.03	6.43 ± 1.33	0.003
15 min	5.51 ± 1.7	4.01 ± 1.08	0.0001
30 min	1.58 ± 0.99	1.36 ± 0.82	0.18

## Discussion

Inhalational induction is used widely in pediatric cases and cases with anticipated difficult intubation [11]. Low blood and tissue solubility and cardiorespiratory stabilizing properties have made sevoflurane a suitable alternative to halothane in children [12]. But compared to halothane, sevoflurane is more widely associated with emergence agitation in children [13]. Even though self-limiting, emergence agitation can lead to bodily injury (of the patient and the caregiver), dislodgement of drains or catheters, and complex postoperative management and patient monitoring [14]. Our study compared fentanyl and dexmedetomidine to prevent emergence agitation after sevoflurane anaesthesia in children.

We recruited 120 children in our study and randomized them into Group F (those who received fentanyl) and Group D (those who received dexmedetomidine). Our study showed a significant difference between the groups concerning their demographic parameters (age, sex, and weight). The different surgical procedures like circumcision, inguinal hernia repair, and orchidopexy were comparable between the groups. Also, there was no difference between the groups concerning their duration of anaesthesia.

The baseline preoperative heart rate of children in Groups F and D was 107.57±13.25 beats/min and 106.42±12.26 beats/min, respectively, and was statistically insignificant. There was a statistically significant decrease in the intraoperative mean heart rate amongst children of Group D compared to the children of Group F at 0 minutes, 10 minutes, and 20 minutes. Towards the later part of the intraoperative period, i.e., at 30 minutes and 40 minutes, the decrease in heart rate was comparable and insignificant. We also found that both groups' heart rates began to rise from 0 to 30 minutes in the postoperative period. However, the postoperative variability in heart rate was also comparable and statistically insignificant.

The baseline preoperative MAP was 77.03 ± 7.9 mmHg for Group F and 78.88 ± 9.97 mmHg for Group D. This difference was statistically insignificant. The MAP decreased significantly amongst Group D children compared to Group F children from 0 to 20 minutes. However, towards the later part of the intraoperative period, i.e., at 30 minutes and 40 minutes, the MAP was comparable between the groups. We also found that in the postoperative period, the MAP began to rise in both groups of children from 0 to 30 minutes. But both the groups were comparable, with no statistically significant difference between them.

Ibacache et al. studied the effect of two different doses of dexmedetomidine on the recovery characteristics after sevoflurane anaesthesia and found that the decrease in heart rate was more in the dexmedetomidine group compared to the placebo group [15]. Patel et al. found that the mean heart rate and mean systolic B.P. were significantly lower amongst children in the dexmedetomidine group than in the fentanyl group during the first 60 minutes [16]. Asaad et al., while comparing the emergence characteristics in ninety patients after sevoflurane anaesthesia, found a more significant decrease in intraoperative heart rate in dexmedetomidine when compared to the fentanyl group (p < 0.05) [17]. This hemodynamic change is similar to our observation. These studies were similar to our finding of a lower heart rate in the dexmedetomidine group compared to the fentanyl group during the intraoperative period.

The mean time to eye-opening was significantly earlier for the children of Group F compared to Group D (9.3 ± 1.1 min versus 10.12 ±1.05 min, p <0.0001). Also, the mean time of shifting to the ward was significantly earlier for the children of Group F than for Group D (17.99 ± 1.95 min versus 19.38 ± 1.63 min, p <0.0001). Guler et al. found that the time to emergence and extubation was significantly longer in the dexmedetomidine group (P < 0.05) when compared to a placebo group after receiving sevoflurane anaesthesia which is similar to our finding [18]. Patel et al. found the time to awake to be earlier for the dexmedetomidine group compared to the fentanyl group. 

This did not tally with our result, probably due to the difference in doses of drugs and the methodology used in our study [16]. Asaad et al. found the time to eye-opening and the time of shifting to the ward to be earlier in the fentanyl group compared to the dexmedetomidine group, which was also similar to our study [17]. Shi did a meta-analysis and found that the time in the PACU in the fentanyl group was longer than that in the control group [19]. In our study, there was no control group, so this cannot be commented upon, but the fentanyl group had a shorter PACU stay than the dexmedetomidine group.

Though the mean time to eye-opening was significant between the groups in our study, the actual difference was 50-180 seconds. Similarly, the mean time to shift to the ward, though significant in our case, was different by 1-4 minutes. These differences, in a clinical sense, don't make much difference.

In our study, the mean CHEOPS score (and hence the pain) was statistically significant between the two groups of children at 0 minutes, with the children in Group D having higher scores than Group F. None of the children had a CHEOP score of 10. Hence rescue fentanyl was not necessary in any of the cases. This is similar to the study by Asaad et al., who found that pain or discomfort was higher in the dexmedetomidine group than in the fentanyl group [17]. Patel et al. also found a higher pain score in the dexmedetomidine group compared to children in the fentanyl group [16].

In our study, the mean Modified Aldrete Score was statistically insignificant between the two groups of children at 0 minutes, while as the duration progressed to 15 minutes, the children in Group F had significantly higher scores than the children in Group D. Thus, the children in Group F recovered from anaesthesia earlier than children of Group D. However, as the postoperative duration increased to 30 minutes, the recovery scores between the two groups of children became insignificant and comparable.

The mean PEAD score was significantly lower in the children of group D compared to children of Group F at 0 minutes and 15 minutes, suggesting that emergence agitation was lesser in frequency in the children of Group D. However, as the duration of the postoperative period increased and went onto 30 minutes, the PEAD scores between the two groups became insignificant and hence comparable.

Patel et al. found that the dexmedetomidine group had a statistically lower frequency of severe emergence agitation than the fentanyl group until 30 minutes postoperatively, after which there was no incidence of extreme emergence agitation in the dexmedetomidine group. However, children in the fentanyl group had an incidence of 1.6% of severe emergence agitation, which corresponds to the result of our study [16]. Contrary to our study, Asaad et al. found a higher incidence of agitation in the fentanyl group compared to the dexmedetomidine group, but the difference was not statistically significant [17]. Also, they failed to mention the exact time at which they took the scores, so a proper comparison could not be made regarding the onset and duration of episodes of emergence agitation.

Limitations of the study

Our study has a few limitations. The sample size was small. Only infra-umbilical surgeries were included in the study. The duration of anaesthesia was also small. We excluded children less than two years or older than eight years. We also excluded children of ASA grade III and above. We also excluded emergency surgeries.

## Conclusions

Emergence agitation is an undesirable side effect of sevoflurane anaesthesia that can affect postoperative monitoring and management. We have conducted a randomized controlled trial and found that dexmedetomidine (0.15 µ g/kg) or fentanyl (1 µ g/kg) administered prophylactically was able to prevent emergence agitation. Although emergence agitation was higher amongst children receiving fentanyl than those receiving dexmedetomidine during the early recovery period, it decreased as the postoperative period increased to 30 minutes. However, more extensive trials and more patients will help us arrive at a more definite conclusion.
